# A Commentary on “Comparing Operationalizations of Eating Disorder Recovery Using a Comprehensive Lens: Physical, Behavioral, and Cognitive Domains” by Bardone‐Cone et al.

**DOI:** 10.1002/eat.70076

**Published:** 2026-03-09

**Authors:** Charlotte Bovenberg, Suman Ambwani, Valentina Cardi, Janet Treasure

**Affiliations:** ^1^ Centre for Research in Eating and Weight Disorders, Department of Psychological Medicine Institute of Psychiatry, Psychology and Neuroscience, King's College London London UK; ^2^ DIS Study Abroad in Scandinavia Copenhagen Denmark; ^3^ Department of Psychology University of Padova Padova Italy; ^4^ South London and Maudsley NHS Foundation Trust, Bethlem Royal Hospital Beckenham UK

**Keywords:** anorexia nervosa, eating disorders, recovery

## Abstract

Defining recovery in eating disorders remains a major challenge due to the absence of standardized, empirically validated criteria. Bardone‐Cone et al. (2025) address this gap by testing multidimensional, transdiagnostic recovery criteria spanning physical, behavioral, and cognitive domains. This commentary evaluates the suitability of these criteria for anorexia nervosa (AN). Applying a partial version of the proposed criteria to two independent AN datasets, a high‐severity inpatient/day‐patient sample and an outpatient sample, revealed very low rates of full recovery and marked instability over time. These findings suggest that, when applied to AN, the criteria may be overly restrictive and insufficiently sensitive to clinically meaningful change, defining recovery as rare and fragile. Three key implications emerge: AN recovery definitions should incorporate broader functional outcomes such as social functioning and quality of life; lived experience perspectives are essential for capturing subjective and identity‐related aspects of recovery; and recovery should be conceptualized as a nonlinear, evolving process rather than a binary state. Although Bardone‐Cone et al.'s work represents an important step toward standardizing recovery definitions, further refinement is needed to ensure that recovery criteria are clinically meaningful and diagnosis‐sensitive for AN.

**Abbreviations**
AN: anorexia nervosa
BED: binge eating disorder
BMI: body mass index
BN: bulimia nervosa
ED: eating disorder
EDE‐Q: Eating Disorder Examination Questionnaire

Longitudinal outcome studies published in recent years have made substantial contributions to understanding long‐term recovery trajectories in eating disorders (EDs), often spanning decades of follow‐up. Despite the significance of this work, the field has long lacked consensus on how recovery in EDs is defined. Unlike disorders such as major depressive disorder or schizophrenia, EDs do not have standardized recovery criteria, creating major challenges for comparing treatment outcomes across studies. In this context, the paper by Bardone‐Cone et al. (2025) offers a valuable contribution by taking a clear, empirical approach to testing different combinations of recovery criteria spanning three dimensions: behavioral, cognitive, and physical. By empirically examining multiple recovery combinations, this work provides an important foundation for future research aimed at refining and validating recovery criteria across EDs. Their framework is transdiagnostic, whereas earlier approaches tend to be disorder‐specific. One of the earliest multidimensional approaches to defining anorexia nervosa (AN) recovery was the Morgan & Russell scale, which assessed food intake, menstrual status, mental state, psychosexual functioning, and socioeconomic functioning (Morgan and Hayward 1988). Notably, its inclusion of social functioning, absent from Bardone‐Cone's criteria, addressed an important element of AN, as social withdrawal is a defining feature of the illness (Stockford et al. 2019).

The authors advocate for transdiagnostic recovery criteria, arguing that this approach reflects the frequent diagnostic crossover and shared recovery processes observed across EDs. While some aspects of recovery may be shared across EDs (subjective perception of recovery status), others are diagnosis‐specific and cannot be meaningfully applied to all EDs. Importantly, not all EDs involve a desire for thinness or body dissatisfaction. Disorders such as avoidant/restrictive food intake disorder and pica are not driven by weight‐ or shape‐related concerns, making cognition‐based measures like the Eating Disorder Examination Questionnaire (EDE‐Q) poor indicators of recovery for these groups. The value of shared physical recovery criteria, in this case a body mass index (BMI) threshold of 18.5, is equally questionable, given the markedly different phenotypes of AN, binge eating disorder (BED), and bulimia nervosa (BN). The same is true for atypical AN, where individuals may experience severe psychological and physical symptoms despite having a BMI in the normal or high range. In these cases, weight suppression and recent weight loss may be more informative than BMI alone. Overall, the wide variation in ED presentations suggests that fully transdiagnostic recovery criteria are unlikely to capture recovery accurately across all diagnoses.

Transdiagnostic recovery criteria are only meaningful if they can validly assess recovery across all ED diagnoses. However, this question of transdiagnostic accuracy remains unanswered. Bardone‐Cone et al.'s study only reports detailed information on the stability and accuracy of their criteria for the total sample (AN [75.8%], BN [47.5%], BED [10.8%], and several other specified feeding or ED presentations [ranging from 3.7% to 33.2%]), not for specific diagnostic groups. While this is a natural limitation of an exploratory study, future research should build on this work by testing whether the proposed transdiagnostic criteria perform consistently across individual ED subtypes. Such work would clarify whether truly shared recovery criteria are both feasible and clinically meaningful across the full spectrum of ED presentations.

To explore the performance of these criteria in AN specifically, we analyzed two independent AN datasets: a high‐severity inpatient/day‐patient sample (Cardi et al. 2024) and an outpatient sample (Cardi et al. 2020). In both trials, the adjunctive interventions (ECHOMANTRA; RecoveryMANTRA) did not significantly outperform treatment as usual (TAU), indicating that the samples are broadly representative of UK‐based standard inpatient/day‐patient and outpatient AN treatment. Detailed descriptions of both samples, including recruitment and trial procedures and sample characteristics, have been published previously (Cardi et al. 2020, 2024). The datasets constrained our ability to evaluate the full criteria. Bardone‐Cone et al. require an absence of an ED diagnosis, which they assessed via the Structured Clinical Interview for DSM Disorders; neither of the datasets examined here provides such information. The behavioral criterion was measured using the EDE‐Q, and follow‐up assessments occurred at 6, 12, and 18 months. As the EDE‐Q only captures behaviors over the previous month, we could not directly assess the required 3‐month absence of binge‐eating, purging, and restricting behaviors. We therefore defined full recovery according to the Bardone‐Cone criteria for full physical (BMI ≥ 18.5 kg/m^2^) and cognitive (all four EDE‐Q subscales within one standard deviation of gender and age‐matched community norms) recovery, but amended their behavioral criterion to a 1‐month absence of binging, purging, and restricting. In line with Bardone‐Cone, partial recovery was defined as fulfilling two out of three criteria.

The observed rates of full recovery and the stability of recovery over time were very low in both samples. In the inpatient sample (*n* = 370), seven participants met full recovery criteria at 6 months after in‐ or day‐patient treatment, six met criteria at 12 months, and only two did so at 18 months. No participant maintained full recovery across all follow‐up assessments. Although four individuals remained fully recovered between 6 and 12 months, none of those who achieved full recovery at 12 months continued to meet criteria at 18 months. One participant who was fully recovered at both 6 and 12 months did not provide 18‐month data, possibly indicating withdrawal from the study after achieving recovery. Partial recovery was more common but did not appear to lead to full recovery: 28 participants were partially recovered at 6 months, yet none reached full recovery at 12 months; similarly, among the 36 participants who were partially recovered at 12 months, only one achieved full recovery by 18 months.

A similar pattern was observed in the outpatient sample (*n* = 187). Seven participants met full recovery criteria at 6 months and nine at 12 months, but only one maintained full recovery across both time points. No participants who were fully recovered at 6 months were lost to follow‐up. Six participants were partially recovered at 6 months, but none progressed to full recovery by the 12‐month follow‐up.

These findings suggest that recovery was both rare and unstable among patients receiving outpatient and inpatient/day‐patient care for AN when using a partial version of the Bardone‐Cone criteria. While some of the observed instability could be attributed to our slight amendment of the behavioral criterion, and the low number of recovered patients limits interpretability, the consistent pattern observed across two independent samples lends credibility to these findings. Taken together, these results raise concern that, when applied to AN samples, the proposed recovery criteria do not identify a stable group of recovered individuals; instead, they appear to define recovery as rare and fragile, potentially underestimating patients' recovery efforts and achievements and undermining perceptions of progress and hope.

We propose three amendments to the definition of recovery, specifically for AN. First, recovery definitions should extend beyond weight, behavior, and ED cognitions to include broader indicators of functioning, such as social functioning and quality of life. A return to a more multidimensional definition, such as that offered by the Morgan & Russell scale, would allow for a more comprehensive view of recovery.

Second, definitions of recovery must incorporate the perspectives of individuals with lived experience, particularly regarding identity and psychological well‐being. While empirical evidence is important when defining concepts such as recovery, it is not sufficient by itself. Patient and public involvement is crucial to ensure that definitions reflect the lived realities of those navigating recovery, including the nonlinear nature of behavioral change and the personal significance of progress that may not align neatly with rigid thresholds. Engaging individuals with lived experience could help identify which aspects of recovery they view as essential, which behaviors or cognitive patterns they regard as acceptable fluctuations, and how best to capture recovery in a way that feels both realistic and validating. Without such input, recovery criteria risk becoming scientifically rigorous yet disconnected from patient priorities and the complexities of real‐world recovery trajectories. For instance, integration of qualitative, patient‐reported outcomes alongside objective measures would allow us to determine whether those deemed “recovered” by quantitative criteria also feel psychologically recovered. EDs are deeply intertwined with identity, control, and emotion, meaning that behavioral and cognitive change does not necessarily equate to a subjective sense of recovery. People with lived experience have stated that regaining an identity outside of their AN and facing their fears of what living without it would look like is an important turning point in their recovery journey (Stockford et al. 2019). These experiential distinctions are, therefore, crucial for understanding the quality and sustainability of recovery, yet remain insufficiently represented in current definitions.

Finally, recovery should be conceptualized as an ongoing, heterogeneous, and nonlinear process rather than a binary outcome. One limitation of the current criteria is the lack of consideration of psychiatric comorbidity. High rates of mood, anxiety, obsessive‐compulsive, and autism spectrum disorders are well documented in AN, which can shape recovery trajectories independently of ED psychopathology. Social difficulties, ritualistic behaviors, and obsessive‐compulsive traits may persist even when ED symptoms have remitted, complicating the notion of a discrete recovered state. This highlights the need for recovery definitions that acknowledge variability rather than assuming uniform endpoints. More broadly, recognizing recovery as an evolving process is particularly salient given the developmental disruption associated with early‐onset AN. Emerging during adolescence, a critical period for identity formation and brain development, the illness can profoundly shape self‐concept and emotional development. For some, the term *recovery* may be misaligned with their lived experience, preferring the term *discovery*, particularly after long‐term illness. Although standardized criteria are vital for research and treatment evaluation, the field may need to adopt more nuanced language, distinguishing between clinical recovery, personal recovery, and developmental repair.

Importantly, the term “recovery” is conceptually and emotionally loaded. In research contexts, it typically denotes sustained remission of Diagnostic and Statistical Manual of Mental Disorders‐defined symptoms and low risk of relapse, a state that is measurable and operationalizable. For patients, however, recovery often signifies something broader: restored agency, identity, relationships, and meaningful participation in life (Stockford et al. 2019). Difficulties arise when these definitions diverge. Individuals who are functioning well and largely free of ED behaviors may nevertheless be told they are not fully “recovered” because they do not meet strict research criteria. This can feel invalidating and demoralizing, and raises an important question: who defines recovery, researchers, clinicians, or those with lived experience? There is clear value in identifying and studying durable symptom remission. However, it may be worth reconsidering whether “recovery” is the most appropriate term for that construct. Greater precision in language could preserve scientific rigor while reducing unintended harm.

Bardone‐Cone et al.'s work makes a valuable and necessary contribution by empirically testing multidimensional, transdiagnostic definitions of recovery. It represents a step toward a more holistic and standardized approach to defining recovery in EDs. At the same time, their work underscores an important conceptual issue: the term “recovery” is not neutral. When defined strictly in terms of sustained symptom remission, it may inadvertently exclude individuals who are faring well both behaviorally and psychosocially, and who experience themselves as meaningfully changed. Greater clarity in distinguishing between clinical remission and personal recovery, or perhaps discovery, may therefore strengthen both research precision and patient‐centered care. Moving forward, the field would benefit from building on this framework by viewing recovery as an ongoing process and incorporating patients' subjective perspectives as an equally valid and essential marker of recovery. Because EDs are so deeply tied to identity and emotion, the patient's own experience is not just an adjunct to recovery research; it is central to understanding what recovery truly means.

## Author Contributions

**Janet Treasure:** conceptualization, writing – review and editing, funding acquisition, investigation. **Suman Ambwani:** writing – review and editing, funding acquisition, investigation. **Charlotte Bovenberg:** conceptualization, writing – original draft, writing – review and editing, formal analysis. **Valentina Cardi:** writing – review and editing, funding acquisition, investigation.

## Use of Artificial Intelligence

AI was not used in any stage of the creation of this commentary.

## Involvement of Person with Lived Experience

Persons with lived experience were not involved in the study design or execution, or in the preparation of this manuscript.

## Funding

The TRIANGLE trial was funded by the National Institute for Health and Care Research (NIHR), under the Health Technology Assessment (HTA) Programme (Grant Reference Number 14/68/09). The views expressed are those of the author(s) and not necessarily those of the NIHR or the Department of Health and Social Care. J.T. received salary support from the NIHR Maudsley Biomedical Research Centre (BRC) at South London and Maudsley (SLaM) National Health Service (NHS) Foundation Trust.

The outpatient trial was funded by the NIHR under its Research for Patient Benefit (RfPB) Programme (Grant Reference Number PB‐PG‐0712‐28041). The views expressed are those of the author(s) and not necessarily those of the NHS, the NIHR, or the Department of Health. J.T. acknowledges financial support from the NIHR Specialist Biomedical Research Centre for Mental Health award to the SLaM NHS Foundation Trust.

## Conflicts of Interest

The authors declare no conflicts of interest.

## Data Availability

The data that support the findings of this study are available from J.T. and V.C. upon reasonable request.
